# Clinical and molecular genetic characterization of familial *MECP2* duplication syndrome in a Chinese family

**DOI:** 10.1186/s12881-017-0486-4

**Published:** 2017-11-15

**Authors:** Xiaoyan Li, Hua Xie, Qian Chen, Xiongying Yu, Zhaoshi Yi, Erzhen Li, Ting Zhang, Jian Wang, Jianmin Zhong, Xiaoli Chen

**Affiliations:** 1 Department of Neurology, Jiangxi Children’s Hospital, Yangming Road, Donghu District, Nanchang, 330006 China; 2 Beijing Municipal Key Laboratory of Child Development and Nutriomics, Capital Institute of Pediatrics, Room 616, NO. 2, Yabao Road, Chaoyang District, Beijing, 100020 China; 3 Department of Medical Genetics, Capital Institute of Pediatrics, Beijing, China; 4 Department of Neurology, Affiliated Children’s Hospital of Capital Institute of Pediatrics, Beijing, China; 5 Department of Laboratory Medicine, Shanghai Children’s Medical Center, Shanghai Jiaotong University School of Medicine, Shanghai, China

**Keywords:** Clinical and molecular genetic characterization, Familial *MECP2* duplication syndrome, Genomic recombination

## Abstract

**Background:**

Chromosomal duplication at the Xq28 region including the *MECP2* gene, share consistent clinical phenotypes and a distinct facial phenotype known as *MECP2* duplication syndrome. The typical clinical features include infantile hypotonia, mild dysmorphic features, a broad range of neurodevelopmental disorders, recurrent infections, and progressive spasticity.

**Methods:**

This Chinese *MECP2* duplication syndrome family includes six patients (five males and one female), and four asymptomatic female carriers. Two kinds of chips including 4x180K CNV + SNP chip and custom 8x60K CNV chip were used to detect *MECP2* duplication, and then fluorescent in situ hybridization (FISH) analysis was performed to identify the exact copy number of *MECP2*. X-chromosome inactivation (XCI) analysis on AR gene was detected for all female family members, and the microsatellite analysis on *MECP2* was used to validate the recombination event on *MECP2* region.

**Results:**

The affected male subjects presented with a broad range of neurodevelopmental symptoms (severe intellectual disability, developmental delay, seizure, language deficit, and autism spectrum disorder) as well as facial dysmorphism and other symptoms which were consistent with that of Western patients previous reported. Seizure is reported in Chinese patients for the first time. In addition, we validated three recombination events for the *MECP2*-duplication allele during maternal transmission due to X homologous recombination.

**Conclusions:**

We provided the largest known Chinese pedigree with *MECP2* duplication syndrome. The detailed clinical description and molecular genetic characterization in all affected family members further delineate the typical phenotype of this genomic disorder in Chinese population.

**Electronic supplementary material:**

The online version of this article (10.1186/s12881-017-0486-4) contains supplementary material, which is available to authorized users.

## Background

X-linked intellectual disability (XLID) is a group of genetically heterogeneous diseases characterized by cognitive impairment and reduced adaptive skills [1]. XLID can be caused by single nucleotide variants (SNVs) and/or copy number variants (CNVs) on the X chromosome [2]. For example, the loss-of-function mutation of methyl-CpG-binding protein 2 gene (*MECP2*) contributes to Rett syndrome [3], while the increased copy number of *MECP2* contributes to *MECP2* duplication syndrome [4].

With the increased usage of chromosomal microarray or other techniques in clinical diagnostic laboratory, *MECP2* duplication can now be detected quickly. Up to 2015, more than 200 patients with *MECP2* duplication syndrome have been described [5–24]. In China, microarray has been applied for clinical genetic diagnosis only in developed region since 2010 [25–27], the exact diagnosis yield of *MECP2* duplication in neurodevelopmental disorders is unknown. Only sporadic Chinese patient with *MECP2* duplication have been reported before. In this study, we reported familial *MECP2* duplication syndrome in a large Chinese family, in which four male patients and four asymptomatic females were confirmed as carrying *MECP2* duplication. Beside the typical neurodevelopmental symptoms such as severe intellectual disability, developmental delay, poor language skills, and autism spectrum disorder (ASD), the symptom generalized tonic–clonic seizure existed consistently in the four male patients. Major non-neurological symptoms, which include recurrent respiratory infections and constipation, existed in all affected male patients. As the third reported case of *MECP2* duplication syndrome in Chinese patients, the detailed neurodevelopmental trajectory and facial dysmorphism in this study will further delineate clinical description and molecular genetic characterization of *MECP2* duplication syndrome in Chinese. Of note, one male patient in this family was the fifth male patient ever reported to have survived past 25 years old [9, 10, 15].

## Methods

### Array comparative genomic hybridization (aCGH) analysis

Genomic DNA was extracted from peripheral blood for all available family members using the QIAamp DNA Blood Mini Kit (Qiagen, Hilden, Germany). The aCGH was performed according to previously published methods using Agilent Oligonucleotide Microarray [28]. Raw chip data were analyzed via DNA CytoGenomics software (Agilent Technologies Inc., Palo Alto, CA). There are two kinds of aCGH used to detect *MECP2* duplication: 4x180K CNV + SNP chip [28] and custom 8x60K CNV chip. The custom 8x60K CNV chip was designed in particular to detect any single genetic CNV on the known X-linked genes (90 genes) [2]. For 8x60K CNV chip, there are 30,000 probes which were designed to cover the entire genome with an average of 30 kb between adjacent probes, allowing for the detections of 300-500 kb CNV on non-X chromosomes (the validation results had showed that most of recurrent neurodevelopmental disorder-related CNV can be detected from this customized chip. Under manuscript); meanwhile, high-density probes (10,000 probes) were designed for the genomic region of 90 X-linked genes. Then, the probes covering each exon of these X-linked genes were checked one-by-one, ensuring that each exonic region (the exon and its flanking 100 bp) contains at least one probe with an average 100 bp between adjacent probes. Finally, the X chromosome contained 35.57% (21347) of the whole genome probes, and the 90 X-linked genes contained 31.39% (18837) of whole genomic probes. 88% of the 1502 exons in the X-linked genes were covered by at least three probes, and 70% of the 1502 exons were covered by at least four probes. All designs were performed on Agilent SureDesign website (https://earray.chem.agilent.com/suredesign/agilent.com).

### Multiplex Ligand-dependant probe amplification (MLPA) analysis and FISH analysis

MLPA probemix P015 (MRC-Holland, Amsterdam, Holland) was used to test *MECP2* duplication in the remaining family members. Those who are not carrying *MECP2* duplication as confirmed by 244 K chip were chosen as controls. The data was analyzed by Genemarker V5.0.14 (SoftGenetics, State College, PA).

In order to identify the exact copy number of *MECP2*, we performed FISH for the proband (III:16) by standard procedures using SureFISH Xq28 *MECP2* probe (spectrum green, Hg19, ChrX:153,286,406–153,368,945) and SureFISH Xq22.31 STS probe (spectrum red, Hg19, ChrX:7,137,093–7,272,886) (Agilent Technologies Inc., Palo Alto, CA).

### X-chromosome inactivation (XCI) analysis on *AR* and the microsatellite analysis on *MECP2*

XCI was examined using androgen receptor (*AR*) methylation assay with minor modifications [29]. The XCI patterns were classified as random (a ratio between 50:50 and 80:20) or skewed (a ratio higher than 80:20). Two aliquots of 500 ng of genomic DNA were incubated at 37 °C overnight, one with 20 units of the methylation-sensitive restriction enzyme HpaII (NEB, Beverly, USA) and the other without. Both products were then used as templates to amplify the (CAG)n small tandem repeat in exon 1 of *AR* gene.

The microsatellite 23 × GT within the *MECP2* gene region was chosen to differentiate the *MECP2-*duplication allele from the *MECP2*-normal allele. This assay was performed on all available family members. The sequence of forward primer used was FAM 5′-TGAGGACAGCCAGAAGGAGT-3′, and that of the reverse primer used was 5′-ACACCCCTTTCCTTTGTGTG-3′.

The PCR products were separated according to size using 3730xl Genetic Analyzer (Applied Biosystems, Vernon Hills, Illinois, USA) and were analyzed by GeneMapper 4.0 software.

The study was approved by the ethics committee of Capital Institute of Pediatrics and Jiangxi Children’s Hospital. Written informed consent was obtained from the patients’ guardian/parent/next of kin for the publication of this report and any accompanying images. Physical and neurological evaluations were completed by a neurologist and a developmental specialist (i.e. QC, JMZ). IQ was measured by Wechsler Intelligence Scale for Children (WISC), DSM-V was used by the neurologist for ASD diagnosis.

## Results

### Clinical information

**Fig. 1:**
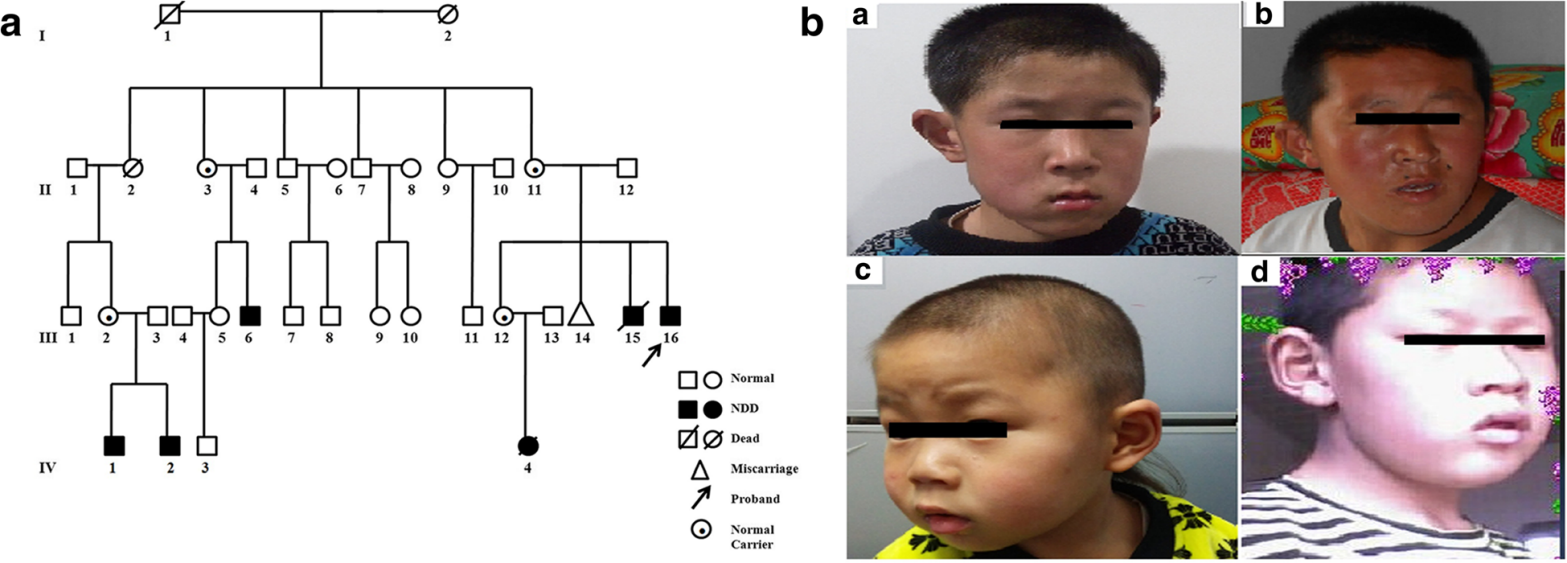
The Chinese family with *MECP2* duplication syndrome. **a** Pedigree of the Chinese family. **b** Facial features of the affected male patients (a. III:16 at 11 years and 2 months old. b. III:6 at 24 years old. c. IV:2 at 2 years and 10 months old. d. IV:1 at 14 years and 8 months old). They show consistent and typical facial features of *MECP2* duplication syndrome, such as midface hypoplasia, long face, down-slanting palpebral fissures, epicanthus, small mouth, jaw protrusion, low-set ears, and large ear lobe and crease

This Chinese family includes a total of six patients with neurodevelopment disorders (five males and one female) from four generations (Fig. 1a). Their physical and neurodevelopmental status were evaluated by a neurologist or a clinical geneticist (Table 1). Four male patients exhibited consistent facial dysmorphism (Fig. 1b). ASD was diagnosed in four male patients (III:6, III:16, IV:1, IV:2) based on the DMS-V criteria.

**Table 1:** The summary of clinical features of the male patients with familial *MECP2* duplication

	Our cases						Literature review (male patients)	
	III:6	III:15	III:16	IV:1	IV:2	%	179	%
Age of enrolled	25 years	NA	11 years and 2 months	14 years and 6 months	2 years and 6 months			
Age of death	–	16 years	–	14 years and 8 months	–			
Intellectual disability	yes	yes	yes	yes	yes	100%	159/160	99.4%
Delayed milestones	yes	yes	yes	yes	yes			
Age of head control	NA	7–8 months	7–8 months	3–4 months	3–4 months			
Age of independent sitting	over 2 years	2 years	2 years	8 months	8 months			
Age learned to walk	over 2 years	over 2 years	over 2 years	3 years	1 year and 6 months			
Feed difficulty	yes	yes	yes	no	no			
Seizure	yes	yes	yes	yes	no	80%	97/162	59.9%
Age for first seizure	7 years	3 years	2 years	10 years	no			
Types of seizures	generalized tonic-clonic seizure, myoclonic seizure, nodding attack and drop attack	generalized tonic–clonic seizures	generalized tonic–clonic seizures	generalized tonic-clonic seizure, myoclonic seizure, nodding attack and drop attack	no			
Hypotonia	yes	yes	yes	yes	yes	100%	141/151	93.4%
ASD or autistic feature	yes	yes	yes	yes	yes	100%	30/41	73.2%
Poor or no eye-to-eye Contact	yes	yes	yes	yes	yes			
Delayed develop peer relationships	yes	yes	yes	yes	yes			
Speech lack or delay	yes	yes	yes	yes	yes			
Stereotyped hand Movement	yes	yes	yes	yes	yes			
Lack of social or Emotional reciprocity	yes	yes	yes	yes	yes			
Recurrent respiratory Infection	yes	yes	yes	yes	no	80%	114/157	72.6%
Constipation	yes	yes	yes	yes	yes	100%	39/103	37.9%
*MECP2* duplication diagnosis	MLPA	clinical feature and family-based linkage analysis	array CGH	array CGH	MLPA			

III:16 was the 11-year-old proband born to a healthy non-consanguineous couple and was delivered with polyhydramnios (birth weight was 3900 g, > 90 percentile). He was noted to exhibit developmental delay at the age of 5 months. He raised his head at 8 months old, walked independently at 2 years old, and uttered “Mama” or “Papa” at 3 years old. He had been referred to Jiangxi Children’s Hospital several times due to global developmental delay since he was 6 months old, and then transferred to the Affiliated Children’s Hospital of Capital Institute of Pediatrics at 11 years old for final diagnosis/treatment. Thus far, he cannot speak any comprehensible phrase or run steadily and eye contact is absent. Central hypotonia was noticed at birth. He developed generalized tonic-clonic seizure at 2 years age, which occurs 1–3 times a year and 3–4 min per attack. Developmental regression was noticed after the first seizure attack. His facial dysmorphic features include midfacial hypoplasia, long face, down-slanting palpebral fissures, epicanthus, small mouth, jaw protrusion, low-set ears, and large ear lobes and creases. He experienced recurrent respiratory infections and refractory constipation with bowel movement every 2–4 days. A brain MRI demonstrated corpus callosum dysplasia. Stereotyped movement (hand flapping, looking at his hands, and biting his fingers) was noticed during clinical interview.

III:15 was an older brother of III:16. His delivery and birth was generally normal except for polyhydramnios and low birth weight (2000 g, < 3 centile). He showed developmental delay since he was 7 months old. He could not say “Mama” or “Papa” even after 3 years old. Severe speech deficit was diagnosed at 7 years old because single words were absolutely absent. Since the age of 3, he had suffered from generalized tonic–clonic seizures frequently. His parents recalled that he developed schizophrenia-like symptom (no contact with family members and repetitive behavior) after multiple onsets of seizure. Beside the neurodevelopmental symptoms, truncal ataxia and unusual gestures were noticed by his parents and recorded in his medical history. He died of a central nervous system infection at 16 years old. His blood sample was not available for genetic testing.

III:6 was born to a non-consanguineous couple, and had normal gestation, birth, and neonatal development (birth weight was 3500 g, > 75 percentile). He presented with developmental delay at 7 months old. He sat and walked steadily at 2 years old, and uttered “Mama” and “Papa” at 5 years old. He suffered from multiple types of seizures (generalized tonic-clonic seizure, myoclonic seizure, nodding attack and drop attack) frequently after 7 years old, and his language ability regressed after the seizure attacks. Stereotyped movement, unusual gestures, hypotonia, and similar dysmorphic features as III:16 were also noticed. Recurrent respiratory infections and constipation occurred occasionally during the first 10 years. He was 25 years old at the time of our last clinical interview, and he had elementary self-care ability. However, he cannot communicate socially with others, and lacked eye-contact. His IQ scores was less than 30.

IV:1 was the first boy born to non-consanguineous parents after normal gestation, delivery, and neonatal period (birth weight was 4000 g, > 95 percentile). He showed developmental delay since 12 months old; he could walk independently and babble some non-conscious words at 3 years old. He also suffered from hypotonia, seizures, constipation and recurrent respiratory infections as the above affected patients did. The types of seizures included generalized tonic-clonic seizure, myoclonic seizure, nodding attack and drop attack. Developmental regression was noticed after the seizures. A brain MRI demonstrated hypomyelination in the periventricular white matter. He presented with stereotyped movements and eye contact was absent. At the age of 14 years and 6 months, he suffered from a central nervous system infection and died 2 months later as a result. All medical evaluations and genetic testing have been completed before his death.

IV:2 was a younger brother of IV:1 with normal gestation and birth (birth weight was 3500 g, > 75 percentile). He showed developmental delay since he was 12 months old. For example, he walked with an ataxic gait after 2 years old. Now, at 2 year and 6 months old, he only can say “Mama” and “Papa”. Like his brother and the other male patients in his family, he presented with stereotyped movements, little eye contact, hypotonia, and constipation However, he did not suffer from recurrent respiratory infections, developmental regression or seizures. Young age is the possible explanation. A brain MRI demonstrated periventricular leukodystrophy. The electroencephalogram was normal.

IV:4 was a girl born to non-consanguineous parents at normal birth (birth weight was 3000 g, > 25 percentile). She died of severe pneumonia and myocarditis at the age of 2. According to her parents, during neonatal period, she had a fever with temperature over 39 °C, and her development was significantly delayed compared to that of age-matched peers. She had not raised her head, sit or walked independently at all until her death. Central hypotonia, recurrent respiratory infections and absence of eye contact were recorded in her medical history. She has experienced generalized tonic-clonic seizures since she was 3 months old. A brain MRI before her death demonstrated diffuse low density. No blood sample was available for genetic testing.

### Familial *MECP2* duplication is identified by aCGH, MLPA and FISH analysis

**Fig. 2:**
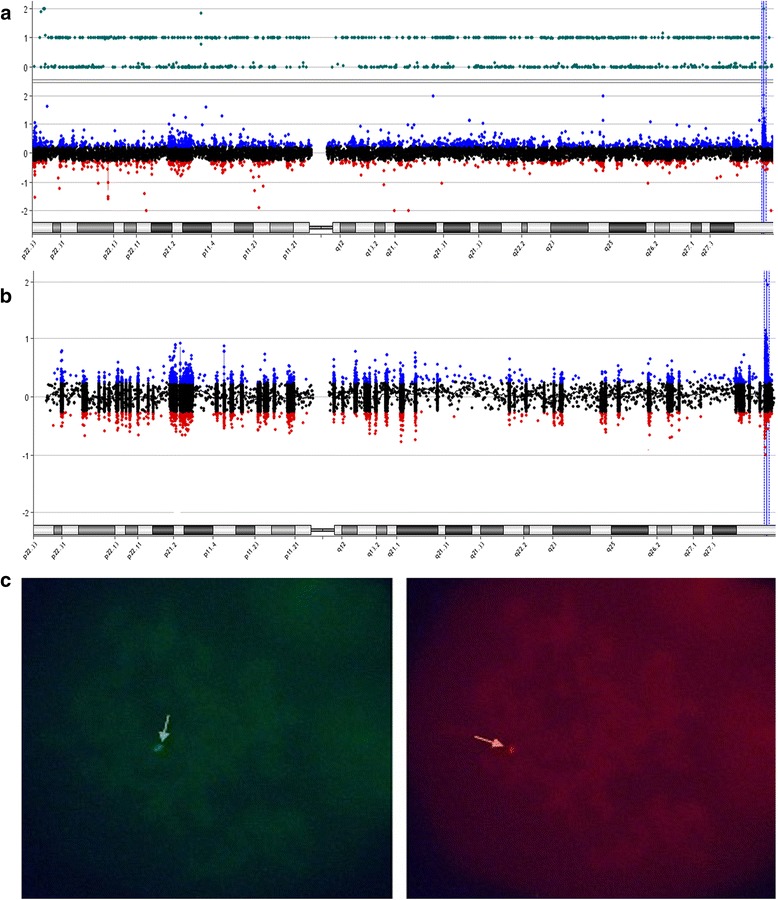
*MECP2* duplication was identified using aCGH or FISH. **a** Result of aCGH (Agilent 4 × 180 K) in patient III:16. It shows a clinically significant 550 kb duplication at Xq28 (chrX:153,056,054–153,606,328 in the hg19). The duplication at Xq28 region contains 24 genes, including *MECP2*, *IRAK1, L1CAM, AVPR2, NAA10, HCFC1*, etc. **b** Results from custom chip (Agilent 8 × 60 K) in IV:1. The ratio (log2 is between 0.5–1.2) suggests that IV:1 carried *MECP2* duplication, not triplication. **c** The result from FISH in III:16. It shows two green signals (Xq28, *MECP2* probe) and one red signal (Xq22.31, STS probe)

The high-density array (4 × 180 K) was used for III:16 only. He showed a clinically significant 550 kb duplication on the Xq28 region (chrX:153,056,054–153,606,328 in the genome build hg19, Fig. 2a). This region contains 24 genes (including *MECP2*, *IRAK1, L1CAM, AVPR2, NAA10, HCFC1,* etc). Afterwards, the custom 8 × 60 K array was used to test for the same *MECP2* duplication in the others. The custom array ultimately showed that the male patient (IV:1) also harbored the *MECP2* duplication (Fig. 2b). Some female family members (II:11, III:2 and III:12) harbored the duplication as well, but others (II:5, II:7, II:9 and III:5) did not. MLPA results confirmed the duplication in patients III:6 and IV:2 and in family member II:3 (Additional file 1: Figure S1). In total, four female carriers (II:3, II:11, III:2 and III:12) and four male patients (III:6, III:16, IV:1 and IV:2) in the pedigree harboured the duplication demonstrating that it was maternally inherited. The dual signal of Xq28 region in patient III:16 confirmed that the patient harbors a *MECP2* duplication rather than a triplication (Fig. 2c). Considering that tandem arrangement is the common form of *MECP2* duplication, we tried to amplify the junction fragment using the breakpoint-specific primers. However, we were unable to map out the sequence feature due to the enriched repeat in this region.

### Skewed XCI patterns and inter-chromosomal homologous recombination during *MECP2* and *AR* region

Analysis of XCI patterns on all the female family members (Additional file 2: Figure S2) showed that the XCI patterns in the female non-carriers were all random (II:9 and III:5), but that of the female carriers were all skewed (II:3, II:11, III:2 and III:12). However, the predominantly inactivated allele of *AR* gene in two female carriers from the same core family (II:3 and II:11) was not the same. II:3 had inactivated maternally inherited allele-279 while II:11 had inactivated maternally inherited allele-285 (Additional file 2: Figure S2B, C). Accordingly, we tested for the alleles of *AR* gene in all male patients (III:6, III:16, IV:1 and IV:2) and found that the male patients did not carry the same allele. More importantly, two male patients from the same core family (brothers IV:1 and IV:2) carried different maternally inherited alleles (IV:1 carried allele-293 and IV:2 carried allele-274, see Additional file 2: Figure S2A). On the basis of above data, we inferred that the inter-chromosomal homologous recombination occurred on the *MECP2-*duplication region.

**Fig. 3:**
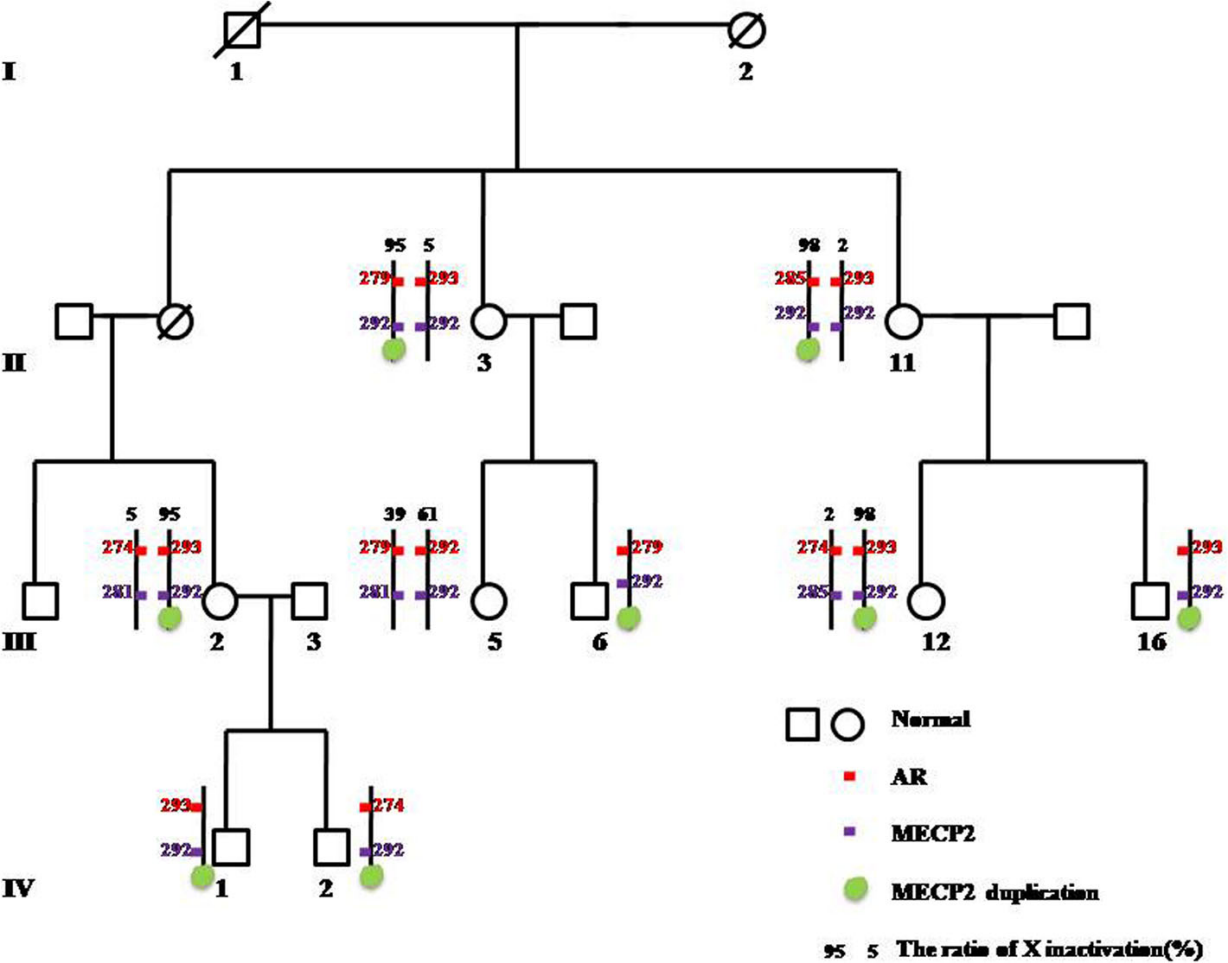
The model for the inter-chromosomal homologous recombination in the *MECP2-*duplication allele

In order to validate the occurrence of homologous recombination in the *MECP2* region, we chose the microsatellite 23 × GT within the *MECP2* gene as the marker for *MECP2* duplication. We found that allele-292 of microsatellite 23 × GT was shared by all of the *MECP2* duplication carriers (Additional file 3: Figure S3), which highlighted the close linkage of *MECP2* duplication to allele-292. Furthermore, after analyzing the relationship between *AR*-specific and *MECP2*-specific microsatellites in every family member, we inferred that the *MECP2-*duplication allele was exchanged with *MECP2*-normal allele by inter-chromosomal homologous recombination during meiosis, resulting in different microsatellite of *AR* gene in the *MECP2-*duplication allele (Fig. 3). This proved that there has been three separate *MECP2* recombination events which occurred during three maternal transmissions (specifically, II:3 and II:11 from I:2; III:5 and III:6 from II:3; and IV:1and IV:2 from III:2).

## Discussion

Methyl-CpG binding protein 2 (MECP2) is a transcriptional repressor that functions by binding directly to methylated CpG dinucleotides and recruiting co-repressor proteins such as histone deacetylases and Sin3a to the promoters of target genes [30, 31]. *MECP2* duplication syndrome refers to the duplication of *MECP2* which is located at Xq28. *MECP2* duplication syndrome was first described in a pedigree with five male patients presenting with severe intellectual disability in 1997 [32]. It was then recognized as a disorder of genomic imbalance using real-time PCR in 2005 [4]. *MECP2* duplication is now recognized as one cause of neurodevelopmental disorders. The genomic copy variant evaluation study from more than 700 patients with unexplained mental retardation suggested the prevalence of the *MECP2* duplication syndrome in male and female patients is approximately 1 and 0.3%, respectively [12].

As a rare and severe genomic imbalance disorder, *MECP2* duplication syndrome is characterized by a broad range of neurodevelopmental abnormalities (severe intellectual disability, developmental delay, seizure, speech delay/deficit, ASD), as well as hypotonia, recurrent respiratory infections, and facial dysmorphism [11]. Recently, Lim et al. examined 56 cases (49 males and 7 females) with *MECP2* duplication syndrome and expanded the phenotypic spectrum of this syndrome, such as scoliosis and gastrointestinal problems [33]. Two Chinese patients with *MECP2* duplication have been reported previously, and they showed that Chinese patients present similarly as patients in western counties [6, 7]. However, these reports have only studied small pedigrees, and do not contain enough information to help Chinese pediatricians distinguish patients with *MECP2* duplication syndrome from patients with other unknown neurodevelopmental disorders. In this study, we described the detailed clinical and molecular genetic characteristics of a Chinese familial *MECP2* duplication. After testing all available members, we identified nine family members with *MECP2* duplication. Although the DNA of patient III:15 was not available for testing, his clinical characteristics and family-based linkage analysis suggested he had *MECP2* duplications syndrome too. It is one of the largest pedigrees of familial *MECP2* duplication, and is the largest Chinese pedigree thus far. This pedigree includes five male patients and four asymptomatic female carriers.

We reviewed 17 related publications on *MECP2* duplication syndrome and analyzed the prevalence of major medical issues from 179 male patients (See Table 1) [6, 7, 9–24]. Core symptoms of *MECP2* duplication syndrome include developmental delay (100%), severe intellectual disability (99.4%), hypotonia (93.4%), ASD or autistic feature (73.2%), recurrent infections (72.6%), and seizure/epilepsy (59.9%). All male patients in this Chinese family presented with the above major symptoms (See Table 1). The consistent neurodevelopmental trajectory and facial dysmorphism in these five male patients further delineated clinical description and molecular genetic characterization of Chinese *MECP2* duplication syndrome. In addition, seizure was reported for the first time in a patient of Chinese Han ethinicity. In particular, four out of the five (4/5, 80%) male patients presented generalized tonic–clonic seizure.

After reviewing all case reports on *MECP2* duplication syndrome published during last 5 years, only four male patients were reported to have survived beyond 25 years [9, 10, 15]. Patient III:6 in our study has survived past 25 years old. He did not present any differently than the patients who have died younger. Continuous clinical interviews and physical/neurodevelopmental evaluations will be performed on him in the future, so that we can learn about the natural pathogenic progression of *MECP2* duplication syndrome. Of note, there have been reports of patients with *MECP2* triplication [18, 34]. The major clinical manifestations of *MECP2* triplication syndrome are similar to that of *MECP2* duplication syndrome except for the additional symptom of macrocephaly. Fittingly, male patients in this study did not present with macrocephaly.

The majority of *MECP2* duplication are inherited, de novo mutations are rare [22, 23]. Most cases are inherited maternally; paternal inheritance is exceedingly rare, and there have been only four reported cases of paternal inheritance, in which the male patients inherited the gene duplication from an unbalanced X/Y translocation [10, 22]. As an X-linked disorder, males are predominantly affected. Females with one copy of *MECP2* duplication are usually asymptomatic carriers as XCI is often skewed and preferentially inactivates the duplication-bearing X chromosome [11]. However, female patients with *MECP2* duplication have been reported sporadically [35, 36]. The molecular genetic pathogenesis of symptomatic female patients included 1) unbalanced translocation between X chromosome and autosome, 2) no skewing of X-chromosome inactivation and 3) skewed XCI where the normal chromosome is preferentially inactivated [35–37]. The symptoms in affected females are the same as those in affected males [2, 10, 12, 14, 38]. In our study, female patient (IV:4) presented with some symptoms of *MECP2* duplication syndrome as the five male patients. She died at the age of 2 years and 10 days. Given the similarities in the clinical presentation and early death, we believe that she was an affected patient although no sample was available for genetic testing. We speculate that the skewed XCI on the normal X chromosome caused her to be symptomatic.

Differential methylation at the 5′ portions of genes on X chromosome is considered to be one of the primary factors involved in XCI [29]. The human *AR* gene is usually selected as the marker for XCI because it is unique in having a (CAG)n repeat in exon 1, and is highly pleomorphic, with a heterozygosity of 90%. However, *AR* gene is located at Xq12, which is 90 Mb away from *MECP2* region, located at Xq28, and the homologous recombination or crossover between the parental alleles can occur during meiosis. As a result of the inter-chromosomal homologous recombination in *MECP2* region, the (CAG)n repeat marker of *AR* gene is not consistent with that of *MECP2-*duplication allele in offspring. Our study confirmed three recombination events for the *MECP2*-duplication allele during maternal transmission due to homologous recombination. Therefore, we should be careful not to ignore inter-chromosomal homologous recombination when studying a gene located on Xq28 (like *MECP2*) while using *AR* gene as a marker for XCI.

## Conclusions

We described the detailed clinical and genetic characterization of a large Chinese family with familial *MECP2* duplication syndrome. The consistency in clinical presentation in affected family members delineate the typical phenotype of this genomic disorder in Chinese population, which include severe intellectual disability, ASD, seizures, respiratory infections, and constipation. Our report will help clinical neurologists recognize the disorder and distinguish it from other unknown neurodevelopment disorders. Meanwhile, although we described the clinical history for each of our patients, the few *MECP2* duplication cases reported from China are insufficient to allow comparison of phenotypes between Asian and Caucasian patients. Collaboration between countries and regions is urgently needed in order to study the natural history of rare diseases such as *MECP2* duplication on a global scale [39].

## Additional files

Additional file 1: Figure S1. The MLPA result of the family members. (PDF 263 kb)

Additional file 2: Figure S2. X-chromosome inactivation results analyzed using (CAG)n STR of *AR* gene. (PDF 221 kb)

Additional file 3: Figure S3. The results for microsatellite 23 × GT of *MECP2* gene. (PDF 182 kb)

## Abbreviations

aCGH: Array comparative genomic hybridization
*AR*: Androgen receptor
ASD: Autism spectrum disorder
CNVs: Copy number variants
FISH: Fluorescent in situ hybridization
*MECP2*: Methyl-CpG-binding protein 2 gene
MLPA: Multiplex Ligand-dependant Probe amplification
SNVs: Single nucleotide variants
XCI: X-Chromosome inactivation
XLID: X-linked intellectual disability
