# Supplementary material for: Clinical and molecular genetic characterization of familial MECP2 duplication syndrome in a Chinese family
Source: BMC Med Genet. 2017 Nov 15;18:131. doi: 10.1186/s12881-017-0486-4 (PMC5688748; doi:10.1186/s12881-017-0486-4)
Supplement: Supplementary file 1 — The MLPA result of the family members. (PDF 263 kb) [file 12881_2017_486_MOESM1_ESM.pdf]

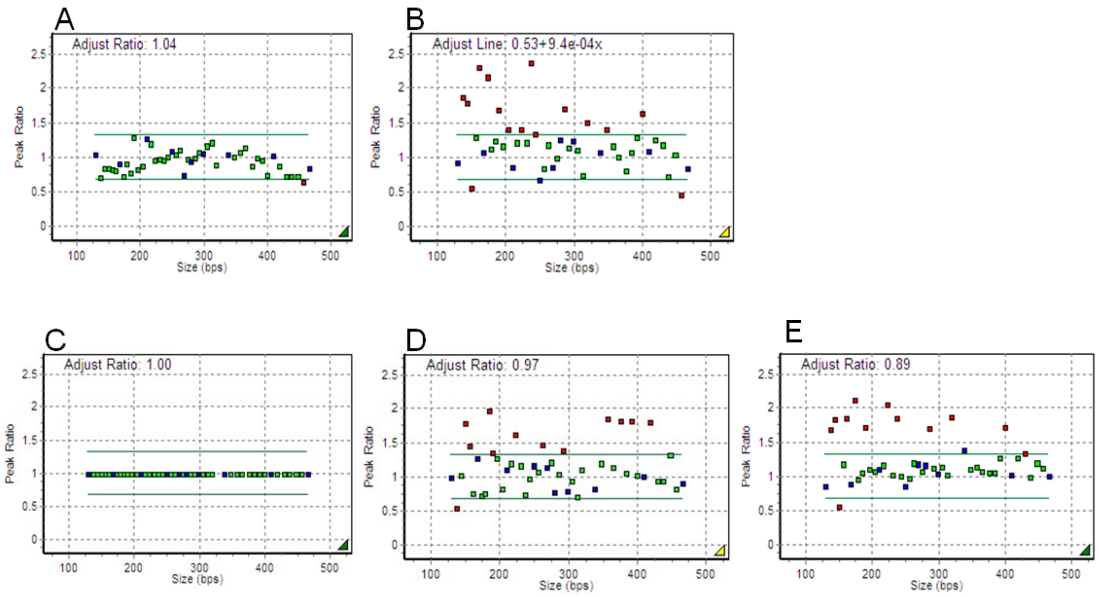

Figure S1. The MLPA result of the family members. A, C show the female and male control, respectively. B, D and E show II:3, IV:2 and III:6, respectively.
