# Supplementary material for: Clinical and molecular genetic characterization of familial MECP2 duplication syndrome in a Chinese family
Source: BMC Med Genet. 2017 Nov 15;18:131. doi: 10.1186/s12881-017-0486-4 (PMC5688748; doi:10.1186/s12881-017-0486-4)
Supplement: Supplementary file 2 — X-chromosome inactivation results analyzed using (CAG)n STR of AR gene. (PDF 221 kb) [file 12881_2017_486_MOESM2_ESM.pdf]

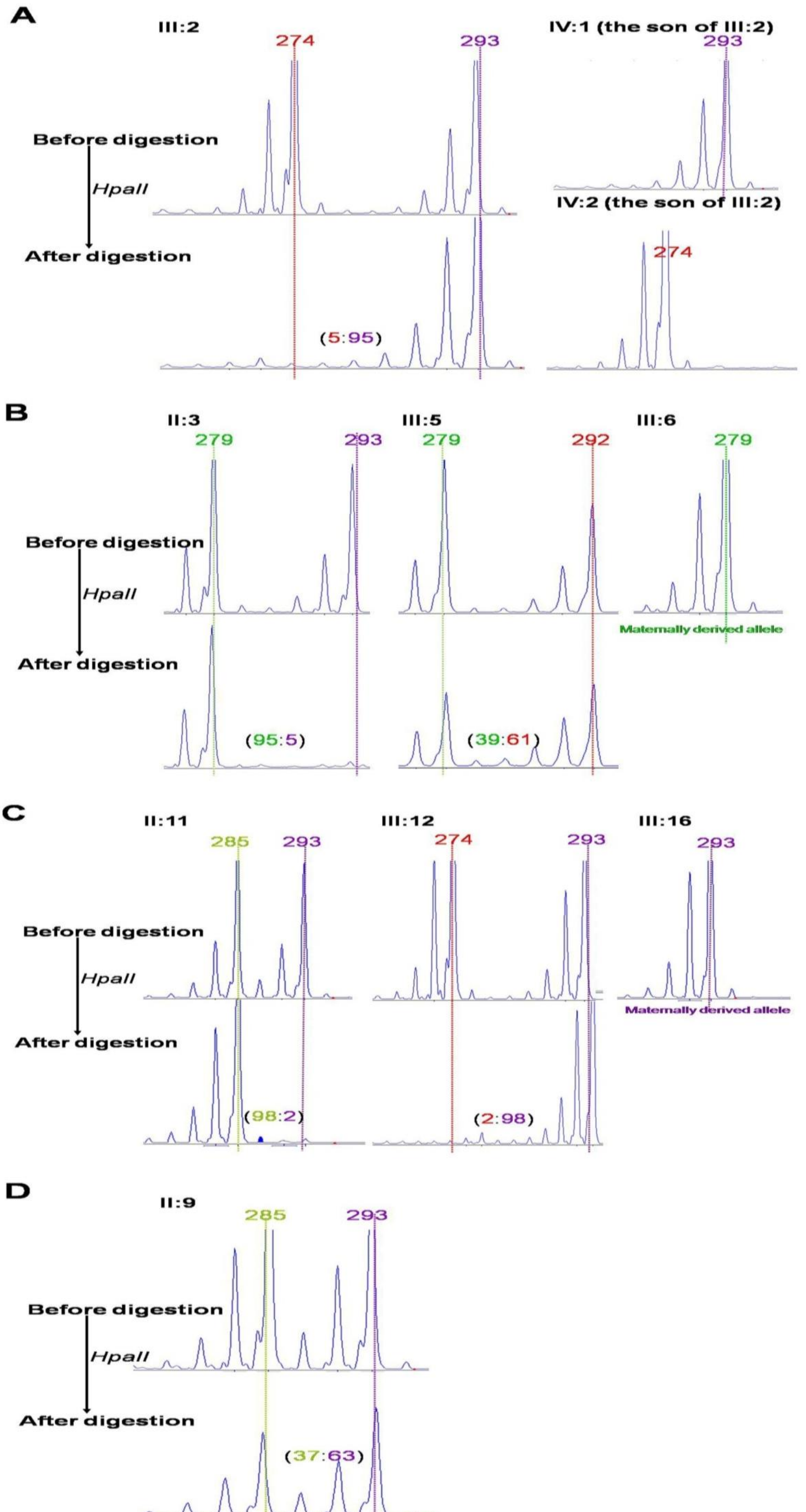
A
III:2
274
293
Before digestion
HpaII
After digestion
(5:95)
IV:1 (the son of III:2)
293
IV:2 (the son of III:2)
274
B
II:3
279
293
(95:5)
III:5
279
292
(39:61)
III:6
279
Maternally derived allele
C
II:11
285
293
(98:2)
III:12
274
293
(2:98)
III:16
293
Maternally derived allele
D
II:9
285
293
(37:63)

Figure S2. X-chromosome inactivation results analyzed using (CAG)n STR of *AR* gene. A shows that the X-chromosome inactivation (XCI) pattern of carrier III:2 is skewed. Her two sons carry the allele-293 and allele-274. B shows that the XCI pattern of II:3 is skewed but the XCI pattern of her daughter III:5 is random (not skewed). C shows that the XCI pattern of II:11 is skewed and that the XCI pattern of her daughter III:12 is also skewed. D shows that the XCI pattern of II:9 is random.
