# Supplementary figures and images for: Clinical and molecular genetic characterization of familial MECP2 duplication syndrome in a Chinese family
Source: BMC Med Genet. 2017 Nov 15;18:131. doi: 10.1186/s12881-017-0486-4 (PMC5688748; doi:10.1186/s12881-017-0486-4)

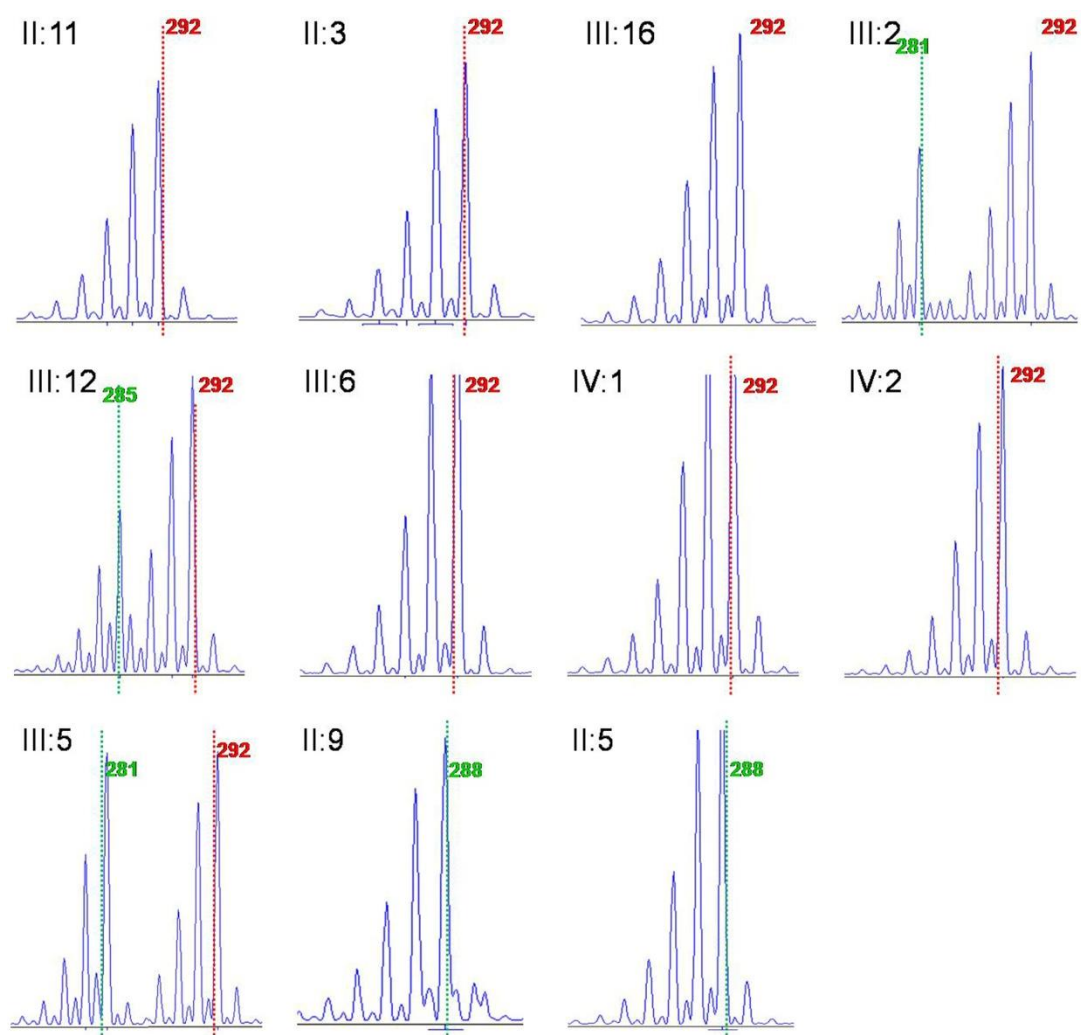

Figure S3. The results for microsatellite 23xGT of *MECP2* gene.

Supplement: Supplementary file 3 — The results for microsatellite 23 × GT of MECP2 gene. (PDF 182 kb) [file 12881_2017_486_MOESM3_ESM.pdf]
